# Recent Advances in the Detection of Aflatoxin M1 in Milk and Dairy Products

**DOI:** 10.3390/bios15120775

**Published:** 2025-11-26

**Authors:** Anna Maria Maurelli, Lucia Catucci, Michelangelo Pascale, Sabato D’Auria, Maria Staiano

**Affiliations:** 1 URT-Bari, Institute of Food Sciences, National Research Council of Italy, Via Orabona 4, 70126 Bari, Italy; annamaria.maurelli@isa.cnr.it; 2 Department of Chemistry, University of Bari, Via Orabona 4, 70126 Bari, Italy; lucia.catucci@uniba.it; 3 Institute of Food Sciences, National Research Council of Italy, Via Roma 64, 83100 Avellino, Italy; michelangelo.pascale@cnr.it (M.P.); maria.staiano@cnr.it (M.S.)

**Keywords:** Aflatoxin M1, biosensors, aptasensors, immunoassays, aptamer-based assay, milk, dairy products

## Abstract

There is an increasing demand to design user-friendly specific assays for the detection of analytes of interest for healthcare, environment, and agrifood. Modern biotechnology has approached this problem by using proteins, enzymes, or RNA/DNA fragments (aptamers) as biological recognition elements for biosensors/assays. The idea is to exploit the extremely wide range of selective affinities sculpted into the various proteins or aptamers by biological evolution. The number of compounds specifically recognized by different proteins and aptamers is very large and ranges from small molecules to macromolecules. The advantages of using proteins and aptamers as molecular recognition elements (MRE) for assays/biosensors are many, and involve relatively low costs in design and synthesis, water solubility, and finally high specificity. Many of the analytes of interest in the food control industry are relatively small. In this case, aptamers and antibodies are widely used as specific MREs in designing advanced biosensors. Aflatoxin B1 (AFB1) is the most frequently found aflatoxin in contaminated food samples, and is one of the most potent natural compounds in terms of genotoxicity and carcinogenicity. Aflatoxin M1 (AFM1) is the hydroxylated metabolite of AFB1 and is usually found in milk and milk products as a carry-over of AFB1 in animals that have ingested contaminated feed. AFM1 is also found in human milk, and has been shown to be hepatotoxic and carcinogenic. Here, we present recent advances in assays and biosensors based on the use of antibodies and aptamers as MREs that have been developed for monitoring the presence of AFM1 in milk and dairy products. The limitations and advantages of aptamer- and antibody-based assays/biosensors are discussed, as well as future research perspectives.

## 1. Introduction

Food contaminants such as aflatoxins (AFs) are one of the main public health problems due to their cancerogenic activity. Aflatoxin M1 (AFM1) is the 4-hydroxylated metabolite of aflatoxin B1 (AFB1), a mycotoxin produced mainly by two ubiquitous fungal species of Aspergillus, i.e., *Aspergillus parasiticus* and *Aspergillus flavus*, frequently co-occurring with aflatoxin B2, G1 and G2 in a large number of commodities intended for human and animal consumption. In addition, the fungal species producing AFs are able to grow on different cereals (i.e., corn, wheat, rice), nuts (i.e., pistachios, peanuts, hazelnuts, almonds), and dried fruits (i.e., dried figs), which spreads their presence in the food chain [1]. AFM1 is secreted in the milk of mammalian species ingesting food or feed contaminated with aflatoxin B1, and has been shown to be resistant to thermal treatments and to pasteurization. For these reasons, AFM1 is commonly found in breast milk, as well as in animal milk and dairy products [2,3].

The toxic effects of aflatoxins have been extensively studied for many years, and it has been shown that aflatoxins are genotoxic and cause liver cancer (hepatocellular carcinoma) in humans and other animal species. Aflatoxins at high doses are also associated with other adverse health effects such as child growth impairment and immune dysfunction [4,5,6,7]. The carcinogenicity of AFM1 has been documented only in experimental animals. Since AFM1 is a metabolite of AFB1, it is presumed to have a toxicity similar to that of AFB1 and to induce liver cancer in rats by a mechanism similar to AFB1. The International Agency on Research on Cancer (IARC) has classified aflatoxins as Group 1 carcinogens, i.e., carcinogenic to humans, and stated that “there is sufficient evidence in experimental animals for the carcinogenicity of naturally occurring mixtures of aflatoxins, and of aflatoxin B1, G1 and M1” [5].

In order to protect human health, several countries have set regulatory limits for the maximum permitted levels of AFM1 in milk [8,9,10,11], ranging from 0.05 μg/kg in the European Union (EU) [9] to 0.5 μg/kg in the United States [10]. In addition, the EU has set lower limits (i.e., 0.025 μg/kg) in infant formulae, follow-on formulae, and young-child formulae, and in food for special medical purposes intended for infants and young children. At present, no maximum permitted levels have been set in the EU for dairy products, although the EU regulation (article 3) states that where no specific EU maximum levels are set out for food which is dried, diluted, or processed, changes in the concentration of the contaminant caused by drying, dilution, or processing shall be taken into account when applying the maximum levels set out for such food. Consequently, AFM1 limits in cheese (or dairy products) should be established according to the processing factor provided by producers. Accordingly, the Italian Ministry of Health has recently proposed four different enrichment factors, ranging from 3 to 6, to set a limit for AFM1 in soft, semi-soft, semi-hard, hard, and very hard cheeses [12].

Several surveys have been carried out worldwide to estimate AFM1 occurrence in milk and relevant human exposure mainly through milk consumption, although additional exposure to AFM1 should be considered due to consumption of dairy products such as cheese and yogurt [2,3,13,14,15,16,17,18]. These studies have highlighted that several nations, mainly in developing countries such as those in South Asia and sub-Saharan Africa, have AFM1 levels in milk higher than EU and US regulatory limits for AFM1, indicating potential risk to humans.

The presence of AFM1 in milk and dairy products is a real risk for human health. Therefore, rapid and reliable methods for the determination of this contaminant in milk and dairy products are necessary both to comply with regulations and to prevent any risk for consumers.

Several liquid chromatographic methods and enzyme-linked immunosorbent assays (ELISA) have been developed for the determination of AFM1 in milk, with the latter used mainly for screening purposes [19,20,21,22,23,24,25]. In particular, a liquid chromatographic method using immunoaffinity column clean-up and fluorescence detection has been validated and adopted as a standard method by the AOAC International for the determination of AFM1 in liquid milk [4]. More recently, liquid chromatography-tandem mass spectrometry (LC-MS/MS) and liquid chromatography-high resolution mass spectrometry (LC-HRMS) are routinely used for the determination of AFM1 in milk and derivative products, such as cheeses and fermented milk products, due to their high sensitivity, selectivity, and the ability to identify and quantify analytes in complex matrices, also allowing for the simultaneous detection of multiple analytes [26,27]. However, these instruments require specialized expertise, and are expensive and time-consuming. Biosensors and immunoassays are becoming increasingly useful tools for rapid detection of food contaminants, including AFM1, because they offer several advantages compared to conventional methods, including high specificity, sensitivity, and portability (allowing on site detection) and are user-friendly. Antibodies and aptamers are the most used biological recognition elements, although other recognition elements such as enzymes, peptides, nanobodies and molecularly imprinted polymers have been explored. In the case of biosensors, these recognition elements are coupled with a transducer that converts the binding event between the recognition elements and the target analyte into a quantifiable signal (optical, electrochemical, thermal, gravimetric). Concerning immunoassays and aptamer-based assays, such as ELISA and lateral flow assays (LFA), the binding event results in a colorimetric or fluorescence signal that can be measured by a spectrophotometer or fluorimeter, or visually in the case of qualitative analysis [28,29,30,31,32].

Many commercially available anti-AFM1 antibodies (poli- and monoclonal) with good selectivity and affinity are available promoting the use of immunoassays and biosensors for AFM1 detection [33,34]. On the contrary, only few AFM1 aptamers have been reported in the literature showing high affinity toward AFM1, although they showed good selectivity towards other mycotoxins [35,36,37].

As far as we are aware, few reviews have been published in the past few years concerning immunoassays [33], electrochemical immunosensors and aptasensors [38,39,40], aptasensors [41], and novel biosensors [34] for AFM1 detection. The present review aims to provide information on recent advances in biosensors and assays based on the use of antibodies and aptamers such as molecular recognition elements for AFM1 in milk and dairy products. The most recent works using antibodies and aptamers are therefore presented into two separate sections, each further subdivided according to the type of signal (optical, electrochemical, etc.). Specifically, Section 2 is dedicated to antibody-based assays/biosensors, whereas Section 3 is dedicated to aptamer-based assays/biosensors. Finally, in the Section 4, the advantages and limitations of these tools are discussed, as well as future research perspectives.

## 2. Antibody-Based Assays/Biosensors for AFM1 Determination

Antibodies (Abs) are proteins belonging to the family of immunoglobulins. Their biological function is to support the immune system by identifying and neutralizing non-self-molecules present in pathogens (e.g., bacteria, viruses, etc.) that penetrate the body. Each individual antibody molecule is able to specifically recognize one or more molecules [42,43] which may possess different size and chemical compositions [44].

It is precisely this characteristic that makes the antibodies specific molecular recognition elements (MREs) in the design of analytical tools of medical, agrifood, and environmental interest (Table 1). In fact, a biosensor must be extremely specific and selective with regard to the target molecule, and this characteristic is ensured by the use of a specific Ab as MRE [45].

In addition to the characteristics of specificity and selectivity, nowadays a biosensor is required to be stable, fast, and above all user-friendly. In fact, it is essential that a biosensor can be used on-site even by non-highly qualified personnel, and can provide a rapid analytical response (even at a level of early warning) [46].

In the agrifood area, user-friendly biosensors represent a valid device for on-the-spot determination of contaminants. However, to date, biosensors based on the use of antibodies are not available on the market. In fact, the large majority of commercial analytical assays based on the use of antibodies for AFM1 quantification are ELISA or LFIA.

### 2.1. Optical Immunosensor for AFM1 Detection

A competitive phosphorescent immunosensor was developed for the quantification of AFM1 in milk, employing quantum dots (QDs) as photoluminescent probes. Two different strategies were assessed: a direct approach, in which QDs were used as secondary antibody markers, and an indirect approach, utilizing a derivative of the AFM1-bovine serum albumin (BSA) antigen (indirect analysis), with the former yielding the best results [47].

**Figure 1:**
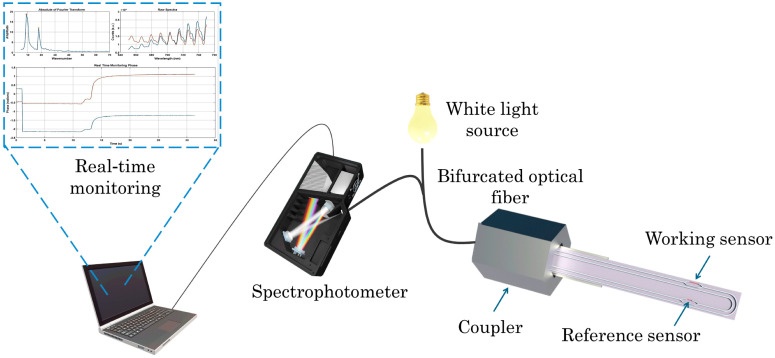
Schematic of the sensor. The chip is composed of two U-shaped silicon nitride waveguides formed as Mach–Zehnder interferometers. Adapted from [48], under Creative Commons Attribution (CC BY) license (https://creativecommons.org/licenses/by/4.0/).

It is worth reporting the work of Kourti et al. [48], who introduced a rapid and sensitive method for detecting AFM1 in milk based on an immersible silicon photonic chip (Figure 1). The device contains two U-shaped silicon nitride waveguides formed as Mach–Zehnder interferometers. One interferometer is functionalized with AFM1-BSA conjugate and the other with BSA alone to serve as a reference. The chip is connected to a broad-band white LED source and a spectrophotometer through a bifurcated optical fiber. The analytical procedure involves immersing the chip into a milk/anti-AFM1 antibody mixture, followed by sequential immersions in in biotinylated anti-rabbit IgG antibody and streptavidin solutions to enhance the signal. The complete assay is accomplished within 20 min, achieving a detection limit for AFM1 of 20 pg/mL in undiluted milk.

Angelopoulou and coworkers proposed a silicon-based optoelectronic immunosensor employing a three-step competitive immunoassay for AFM1 detection. The procedure involves consecutive reactions with a polyclonal anti-AFM1 antibody, a biotinylated polyclonal anti-IgG antibody, and streptavidin [49]. The sensor was tested in milk, chocolate milk, and yogurt with calculated LODs of 0.005 ng/mL for the former matrices, and 0.01 ng/mL for the latter. Interestingly, there was no sample pretreatment for milk analysis.

### 2.2. Strip-Based Immunosensor for AFM1 Detection

Wu and coworkers designed an immunochromatographic test based on the principle of antigen competition for the simultaneous detection of AFM1 and chloramphenicol (CAP) in milk. Specifically, ovalbumin conjugates of the two compounds and goat anti-rabbit IgG were adsorbed onto a membrane to form two test lines (T1 and T2) and a control line (C). For analysis, the strip is immersed in a well containing the sample, together with the AFM1-gold and the CAP-gold conjugates. Focusing on the detection of AFM1, its presence is correlated with the absence of a red line in the T1 zone of the immunological strip. This occurs because at concentrations above a specific threshold, the AFM1 toxins bind to all available AFM1 antibody sites on all the gold nanoparticles, and the nanoparticles, responsible for the red colour, do not bind to the AFM1–ovoalbumin conjugate in the T1 line on the immunostrip [50].

### 2.3. Electrochemical-Based Immunosensor for AFM1 Detection

**Figure 2:**
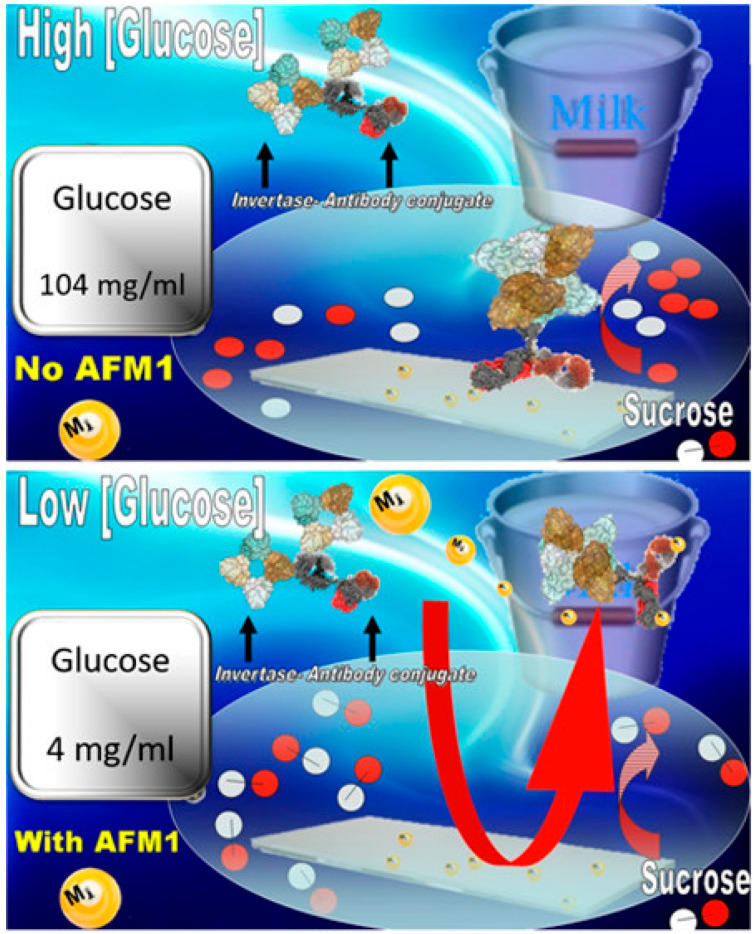
Cartoon representation of the immuno-reaction of conjugate IgGMS-M1-INV on a strip derivatized with Aflatoxin-protein in the absence (**A**) or presence (**B**) of AFM1 in whole milk. Reprinted from [51] (https://doi.org/10.1021/acsomega.9b01300).

Recently, the design of a different method has been reported for rapid detection of AFM1 in milk collected daily by farmers [51]. This method is based on the use of an ad-hoc engineered glucometer device. In particular, an immune detection strip containing invertase conjugated to antibody anti-AFM1 was produced, and a competitive assay was developed. This assay was able to detect the concentration of twenty-seven parts per trillion (ppt) of AFM1 in whole milk (below the EU maximum permitted level) by quantifying the glucose generated by the invertase-conjugated antibody anti-AFM1 strip following one hour of incubation time (Figure 2).

The key innovation of this approach is that the test is based solely on glucose production via an invertase-linked immune-sorbent assay (InLISA) and its monitoring of simple glucose through a commercial glucometer.

Erdil and collaborators proposed a paper-based biosensor device based on a competitive test as an alternative method for the detection of AFM1, which uses magnetic nanoparticles to increase the signal [52]. Moreover, an electrochemical immunosensor based on screen-printed carbon electrodes (SPCE) functionalized with anti-idiotypic nanobodies for the detection of AFM1 was designed [53], showing a linearity range between 0.25 and 5.0 ng/mL, and a detection limit of 0.09 ng/mL. In spiked milk samples, recovery rates increased from 82.0% to 108.0% and RSD values from 10.1% to 13.0%.

**Table 1:** Antibody-based assays/biosensors for AFM1 detection in milk.

Type	Sample Type	LOD (ng/mL)	Recovery (%)	Reference
Optical (Mach–Zehnder interferometer)	milk	0.02 *	n.r.	[48]
	cow milk	n.r.	90.0–107.0	
	sheep milk	n.r.	93.3–107.0	
	goat milk	n.r.	86.7–112.0	
Optical (Mach–Zehnder interferometer)	full-fat milk	0.005	90.0–110.0	[49]
	chocolate milk	0.005	90.0–115.0	
	yogurt	0.01	86.7–106.0	
Immunostrip	optimized conditions	0.1 **	n.r.	[50]
	milk	0.25 **	n.r.	
Electrochemical	whole milk	0.027 *	n.r.	[51]
Electrochemical	optimized conditions	0.09	n.r.	[53]
	milk	n.r.	82.0–108.0	

n.r.: value not reported; * converted to ng/mL; ** cut-off level.

## 3. Aptamer-Based Assays/Biosensors for AFM1 Determination

Aptamers are short synthetic single-stranded nucleotide sequences selected from a randomized library of oligonucleotides through a process known as SELEX (Sequential Evolution of Ligands by Exponential Enrichment). Aptamers have been used as a bio-recognition element in a variety of sensors (Table 2) due to their remarkable characteristics such as low immunogenicity and toxicity, low production cost, high affinity for their targets, and ease of modification [54]. Compared to antibodies, aptamers have lower costs, greater ease of production, higher affinity, and greater chemical and thermal stability [55,56].

### 3.1. Colorimetric-Based Aptasensor for AFM1 Detection

Several technologies in this field exploited the different tendency of AuNPs to aggregate in the presence or absence of the toxin which leads to a colour change, to an extent proportional to the amount of target content. Among these, Kasoju and coworkers developed a paper microfluidic device for AFM1 detection as a convenient alternative for on-site detection technologies [55]. The proposed technology is based on an aptamer/AuNPs complex arising from the physisorption of specific aptamers onto the surface of AuNPs. In the presence of AFM1, the aptamer dissociated from AuNPs, resulting in aggregation and a solution colour change from wine red to blue. The ratio between the absorbance values at 630 and 520 nm was used to determine the aggregation. In addition to the spectroscopic method, the presence of the toxin was detected with the naked eye. The concentration range was from 1 μM to 1 pM, with a LOD of 3 pM and 10 nM in spiked water and milk samples, respectively. Moreover, the device was stable at room temperature for up to 3 months. Another aptasensor based on the principle that AuNPs in NaCl solution do not aggregate when the toxin is present and aggregate in its absence has been proposed. In detail, AuNPs are added to a suspension containing aptamer-modified streptavidin-coated silica nanoparticles, the complementary filament of the aptamer, and the sample to be analysed. In the presence of the toxin, the complementary filament detaches from the silica NPs and stabilises the AuNPs in the presence of NaCl [54]. The quantification was carried out by monitoring the absorbance ratio at 650 and 520 nm. The obtained linear dynamic range was between 300 and 75,000 ng/L, with a LOD of 30 ng/L. In AFM1 spiked milk samples, the low detection limit was 45 ng/L and the recovery rate was between 92% and 109.5%. Tests that incubated the sensor with other toxins, such as OTA, ZEN, DON, and AFB1, showed great specificity toward AFM1 toxin.

Wei et al. prepared a sensor based on the interaction between aptamer-modified AuNPs@CuO and cDNA-Modified Fe_3_O_4_ [57]. They screened the better aptamer by using a combination of a five-segment library and GO-SELEX. With the selected sequence, the assay displayed linearity in the range 0.5–500.0 ng/mL and a detection limit of 0.50 ng/mL. In milk powder the detection recovery was around 92.8–105.2%. For comparison, the recoveries obtained with the ELISA test were investigated, which ranged between 89.20% and 93.10%.

A test strip allowing for visual detection of the AFM1 in the samples was obtained by developing an aptamer-based lateral flow assay (LFA) based on AuNPs [56]. The concentration of AFM1 was inversely proportional to the signal and was given by relative colorimetric signal intensity of AuNPs at the control and test line after 10 min of incubation. For the quantitative analysis, photographs of the test strips were taken and analysed with ImageJ. The linear range was from 0 to 500 ng/mL, and the detection limit was 0.21 ng/mL. The sensor demonstrated that it was specific for AFM1 detection with recoveries in milk samples ranging from 92% to 104.3%.

### 3.2. Surface Plasmon Resonance-Based Aptasensor for AFM1 Detection

A label-free colorimetric aptasensor was developed by Lerdsri et al. by exploiting localized surface plasmon resonance (LSPR) [58]. The sensor exploited competitive interactions of the aptamer to the AFM1 or the AuNPs under a specific condition by using sodium chloride to aggregate AuNPs. In particular, the aptamer interacting selectively with AFM1 changes its structure, and is therefore no longer able to prevent NaCl-induced aggregation of AuNPs that causes a redshift of the LSPR absorption spectrum. The linear response ranged from 0.005 to 0.100 ng/mL, and the detection limit was 0.002 ng/mL. The percentages of recovery obtained in milk samples were in the range of 80.5–89.7%, with an RSD value lower than 10%.

### 3.3. Fluorescence-Based Aptasensor for AFM1 Detection

**Figure 3:**
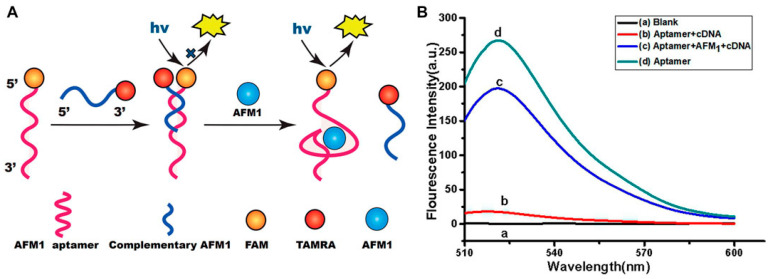
Schematic illustration of (**A**) the aptamer-based fluorescence sensor AFM1 and (**B**) fluorescence emission spectra. Reprinted from [60] under Creative Commons Attribution License (CC BY) (https://creativecommons.org/licenses/by/4.0/).

Technologies based on quenching or modification of the fluorescence signal due to changes in structural conformations have been proposed in several studies, and are recognised as promising for the sensing of biomolecules due to their enhanced sensitivity and specificity [59]. In this perspective, Qiao and collaborators designed an aptasensor based on the generation of fluorescence signals in the presence of the AFM1 toxin [60]. In detail, the AFM1 aptamer was functionalized with carboxyfluorescein, while complementary DNA sequences (cDNA) were implemented with a carboxytetramethylrhodamine group. When AFM1 was not present, the aptamers were hybridized with cDNA, causing fluorescence quenching. In the presence of AFM1, an AFM1/aptamer complex formed, leading to the release of the cDNA and the consequent generation of a fluorescence signal (Figure 3). Under optimized conditions, the sensor displayed linearity from 1 to 100 ng/mL AFM1 concentration and a LOD of 0.5 ng/mL. In milk samples, recoveries from 93.4% to 101.3% were obtained.

Aran et al. developed a fluorescence-based aptasensor which allowed the simultaneous visual detection of AFM1 and chloramphenicol [61]. To this end, a DNA hydrogel was obtained by using an acrydite-modified chloramphenicol aptamer sequence which underwent a gel-to-sol transition in the presence of chloramphenicol. The LOD and LOQ values for AFM1 were 1.7 and 5.2 nM, respectively. The recovery range obtained in milk sample steps was between 91.3% and110.2%.

A multiplexed detection of AFB1 and AFM1 in PBS 1X, milk, and serum was obtained by preparing ternary transition metalsulfide-based PEGylated nanosheets and exploiting the fluorescence turn-on mechanism as a consequence of conformational changes due to the formation of aptamer/toxin complexes [62]. For the AFM1 toxin, a linear response was obtained between 10^−12^ and 5 × 10^−7^ M in PBS 1X, 2.5 × 10^−12^–5 × 10^−7^ M in milk, and 10^−11^− 5 × 10^−7^ M in serum. The LOD values were about 1 pM, 9.87 pM, and 9.59 pM in PBS 1X, milk, and serum, respectively. The recoveries in milk ranged from 96.67% to 101.65%.

Cai and collaborators proposed a sensor for the simultaneous detection of AFB1 and AFM1 toxins by integrating the properties of functionalized graphene oxide and aptamers [63]. The fluorescence resonance energy transfer (FRET) mechanism was exploited for the detection, and a LOD of 8.7 pg/mL for AFB1 and 20.1 pg/mL for AFM1 was obtained. Also, a label-free fluorescent aptasensor for AFB1 and AFM1 detection was obtained by truncating and mutating stem region bases in a 28 nt aptamer, resulting in a LOD of 0.0060 ng/mL and 0.010 ng/mL for the two toxins, respectively [64]. Finally, Naz and collaborators proposed a dual-mode sensor for AFM1 detection by exploiting covalent organic framework-based aptananozymes [65]. The designed architecture allowed for the detection of the presence of the toxin by generating a colorimetric signal, also detectable with the naked eye, and a fluorescent signal, with LOD values of 7 and 5 pg/mL, respectively.

### 3.4. Electrochemical-Based Aptasensor for AFM1 Detection

Electrochemical-based aptasensors involve different techniques, such as cyclic voltammetry (CV), square wave voltammetry (SWV), differential pulse voltammetry (DPV), electrochemical impedance spectroscopy (EIS), and analysis of capacitive signals. Moreover, several technologies in this field relied on the use of NPs. In detail, an electrochemical aptamer-based sensor was developed using an amino-functionalized dendritic fibrous nanosilica (KCC-1-nPr-NH2) and gold nanoparticle supported by chitosan (AuNPs-CS) with a unique toluidine-labelled aflatoxin M1 oligonucleotide docked at the interface [66]. The quantification of AFM1 was achieved by means of CV, SWV and DPV. Square wave voltammetry proved to be the most accurate technique for the determination of AFM1. The linear range was from 10 fM to 0.1 μM, with a lower limit of quantification (LLOQ) of 10 fM. In pasteurized milk spiked with AFM1, the LLOQ for DPV and SWV measurements was 10 fM. The sensor was stable for up to four days.

Hamami et al. proposed a screen-printed carbon electrode aptasensor implemented with AuNPs, ferrocene tetraethylene glycol ligand, and an anti-AFM1 aptamer [67]. Here, the ferrocene was bound to AuNPs and acted as a capacitance transducer, while PEG was effective in preventing non-specific adsorption of biomolecules or microbials. The sensor showed a dynamic range of 20 to 300 pg/mL, with a capacitance signal decreasing with increasing AFM1 concentrations. The LOD was 7.14 pg/mL (S/N = 3). The platform exhibited high selectivity toward AFM1 even in the presence of 1000 folds of interferent toxin concentrations (ochratoxin B and picrotoxin), and the analysis carried out in AFM1 spiked pasteurized cow milk showed recovery percentages in the range of 101.6–105.5%.

Also, an electrospun carbon nanofiber mat was developed for the detection of the AFM1 toxin [68]. Here, the electrode was implemented with AuNPs and thiol-modified single stranded DNA. Cyclic voltammetry was exploited to quantify the toxin. The sensor showed a detection limit of about 0.6 pg/mL and linearity in the range 1–100 pg/mL. Moreover, it displayed good selectivity against AFB1 and AFB2 toxins, good reproducibility, and stability for at least 16 days. Recoveries in milk samples ranged from 106–109%, comparable to HPLC results.

Another electrochemical aptasensor for AFM1 was designed by using Apts-Au@Ag, cDNA2-Au@Ag conjugates, and methylene blue as the electroactive substance [69]. Differential pulse stripping voltammetry was used for the quantification of the toxin. The linear detection range was from 0.05 ng/mL to 200 ng/mL, and the LOD was about 0.02 ng/mL. This sensor also showed good reproducibility, stability, and selectivity. Recoveries in cow, goat, and sheep milk samples ranged from 89.00% to 104.05%, and the RSD ranged from 4.3% to 7.9%.

A label-free electrochemical aptasensor was developed, exploiting a reduced graphene oxide (rGO) and AuNPs-based pencil graphite electrode with the aptamer self-assembled on the surface [70]. The detection was carried out by electrochemical impedance spectroscopy (EIS). The sensor displayed a linear concentration range of 0.5–800 ng/L and LOD of 0.3 ng/L. Stability tests showed that the platform kept 91% of its initial response after 14 days at 4 °C. Analysis performed in raw, low-fat pasteurized, and full-fat pasteurized milk spiked with AFM1 (50 ng/L) showed average recoveries of 92.0%, 108.0%, and 90.0%, and RSD of 5.2%, 4.5%, and 5.7%, respectively.

**Figure 4:**
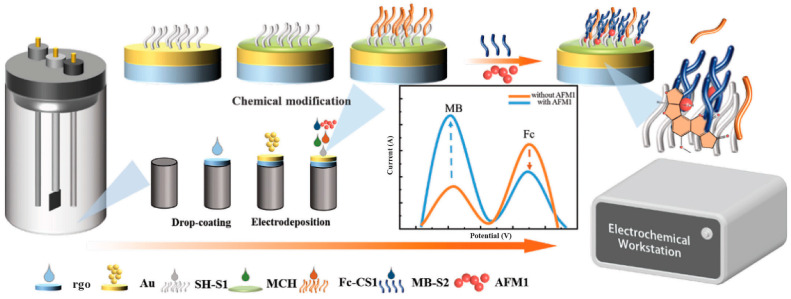
Picture representing the fabrication of a ratiometric electrochemical aptasensor for detection of AFM1. Reprinted from [71] under the CC BY license (http://creativecommons.org/licenses/by/4.0/).

Au-rGO nanomaterials were also used by Li and collaborators [71] to develop a ratiometric electrochemical aptasensor with the AFM1 aptamer split in two portions (S1 and S2), and the square waver voltammetry peak current was monitored for the AFM1 quantification (Figure 4). Specifically, S1 was anchored on the rGO-modified electrode and S2 was modified with methylene blue (S2). A strand complementary to S1 with ferrocene was added. In the presence of the toxin, the complementary strand was released from the electrode surface, leading to a decrease in ferrocene and an increase in the methylene blue signal. They obtained a linear range for the quantification from 0.03 μg/L–2.00 μg/mL and a LOD of 0.015 μg/L, while in milk the linearity was from 0.2 μg/L–1.00 μg/L and the detection limit was 0.05 μg/L.

The molecular imprinting technique (MIT) is useful for preparing molecularly imprinted polymers (MIPs) with cavities that precisely fit the target molecule [72]. In particular, aptamers can be combined with MIPs to fabricate a selective sensor. Yang et al. designed a molecularly imprinted polymer and aptamer based electrochemical sensor with two recognition elements, i.e., aptamer and MIP, to detect AFM1 in milk [73]. The DPV current was then analysed. The platform showed a linear range of 0.01–200 nM and a limit of detection of 0.07 nM (S/N = 3). The stability was about 88% after 21 days, and the recoveries in goat, sheep, and cow milk were in the range of 97.9–105.0%, 95.4–102.1%, and 96.0–105.6%, respectively.

A dual-functionalized electrochemical aptasensor was proposed by Huma and collaborators, made of COOH-functionalized AFM1 aptamer and hydroxyazobenzene polymers at pencil graphite electrodes (PGE) [74]. Hazo-POPs exhibit both electroactive potential and peroxidase activity; thus, two methods have been tested. Method I involved CV measurements and worked in phosphate-buffer aline (PBS) solution, and the PGE was implemented with Hazo-POPs@COOH-Apt to optimize the electrochemical response. Method II employed DPV measurements in acetate buffer and exploited the peroxidase activity of Hazo-POPs. The biosensor showed a linear range from 0.005 to 500 nM, with LODs of 0.004 and 0.003 nM for method I and II, respectively. The recoveries in spiked milk samples were from 101.2% to 104.0%, with RSD values inferior to 3.

An electrochemiluminescence micro-reactor with increased intensity and stability was developed, exploiting the assembly of tris(2,20-bipyridyl) ruthenium(II) onto covalent organic frameworks and used as aptasensor for the detection of AFM1 toxin [75]. The sensor showed a linear response from 0.03 pg/mL to 0.3 mg/mL toxin concentration, and a detection limit of 0.009 pg/mL in optimized conditions, while the recovery in defatted milk was about 93.3–104.0%.

**Table 2:** Aptamer-based assays/biosensors for AFM1 detection in milk.

Type	Sample Type	LOD (ng/mL)	Recovery (%)	Reference
Colorimetric	water	9.8 × 10^−4^ *	n.r.	[55]
	milk	3.28 *	n.r.	
Colorimetric	optimized conditions	0.03 *	n.r.	[54]
	milk	0.045 ng/mL *	92.0–109.5	
Surface plasmon resonance	MOPS buffer containing methanol (10%), pH 7.0	0.002	n.r.	[58]
	milk	n.r.	80.5–89.7	
Colorimetric	binding buffer	0.21	n.r.	[56]
	milk samples	n.r.	92.34–104.35	
Colorimetric	optimized conditions	0.50	n.r.	[57]
	milk powder	n.r.	92.8–105.2	
Fluorescence	optimized conditions	0.5	n.r.	[60]
	milk	n.r.	93.4–101.3	
Fluorescence	optimized conditions	0.56 *	n.r.	[61]
	milk	n.r.	91.3–110.2	
Fluorescence	pbs 1x	3.28 × 10^−4^ *	n.r.	[62]
	milk	8.2 × 10^−4^ *	96.67–101.65	
Fluorescence	optimized conditions	0.010	n.r.	[64]
	raw milk	n.r.	89.0–95.6	
	raw goat milk	n.r.	94.9–112.0	
	pure milk	n.r.	100.0–114.0	
Fluorescence	optimized conditions	0.0201 *	n.r.	[63]
	pure milk	n.r.	97.1–101.0	
Colorimetric fluorescence	optimized conditions	0.007 *	n.r.	[65]
	milk	n.r.	97.0–99.0	
	optimized conditions	0.005 *	n.r.	
	milk	n.r.	96.0–101.0	
Capacitive signal	optimized conditions	0.00714 *	n.r.	[67]
	pasteurized cow milk	n.r.	101.6–105.5	
Cyclic voltammetry	optimized conditions	6 × 10^−4^ *	n.r.	[68]
	milk	n.r.	106–109	
Electrochemical aptasensor	optimized conditions	0.02	n.r.	[69]
	cow, goat, and sheep milk	n.r.	89.00–104.05	
Electrochemical aptasensor	optimized conditions	3 × 10^−4^ *	n.r.	[70]
	raw milk	n.r.	92.0	
	low-fat pasteurized milk	n.r.	108.0	
	full-fat pasteurized milk	n.r.	90.0	
Ratiometric electrochemical aptasensor	optimized conditions	0.015 *	n.r.	[71]
	milk	0.05 *	n.r.	
Electrochemical	optimized conditions	0.023 *	n.r.	[73]
	goat milk	n.r.	97.9–105.0	
	sheep milk	n.r.	95.4–102.1	
	cow milk	n.r.	96.0–105.6	
Electrochemical	method I, in pbs	0.0013 *	n.r.	[74]
	method II, in acetate buffer	0.00098 *	n.r.	
	milk	n.r.	101.2–104.0	
Electrochemiluminescence	optimized conditions	9 × 10^−6^ *	n.r.	[75]
	defatted milk	n.r.	93.3–104.0	

n.r.: value not reported; * converted to ng/mL.

## 4. Conclusions and Future Perspectives

As shown in this review, continuous efforts are being made to develop rapid, low-cost, and reliable immuno- or aptamer-based biosensors and assays for the determination of AFM1 in milk and dairy products.

Table 3 summarizes major advantages and limitations of antibody- and aptamer-based biosensors and assays. Compared to immunosensors, aptasensors have lower cost of production, lower batch-to-batch variability, higher affinity, customizable modification, and chemical and thermal stability. In addition, aptamers offer more flexibility without the ethical issues associated with the production of antibodies in animals.

One of the main challenges in aptasensor development is their limited sensitivity when detecting small molecules, as they typically possess only a single binding site. Additionally, the environmental conditions of real samples differ considerably from those of laboratory buffers, often resulting in non-specific binding and false-positive results. To address these issues, the development of split aptamers, a post-SELEX modification aimed to divide a parent aptamer into smaller functional fragments with high affinity and specificity, has emerged as a promising strategy. Split aptamers are particularly well-suited for detecting small molecules with limited binding sites, offering increased flexibility and precision in sensor applications. This innovative approach has recently been applied to successfully detect some mycotoxins, including AFM1 [76,77].

Despite the great number of advantages of aptamers compared to antibodies, currently, commercially available enzyme-linked immunosorbent assays (ELISA) and lateral flow immunoassays (LFIA) or strip tests are commonly used for fast and quantitative detection of AFM1 in milk samples due to their high specificity, rapid responses, and good sensitivity, and nowadays LFIA is one of the most promising technologies for the rapid and on-site detection of AFM1 in milk. This technology has several advantages that make it particularly useful in the food safety sectors, including being cost-effective, user-friendly and sensitive. LFIA tests do not require sophisticated equipment or highly trained personnel, making them ideal for field monitoring. It is particularly advantageous for rapid testing and real-time screening. However, milk is a complex matrix containing a variety of components that could interfere with the recognition process and cause false positive results, so confirmatory methods (i.e., HPLC-FL or LC-MS/MS) are mandatory. In addition, milk of different origins varies consistently in its composition, and the same sensor/assay developed for one type of milk might not be applicable to other types of milk samples. Preliminary treatment of the sample is often necessary. In case of dairy products, such as cheese and yogurt, an extraction step with organic solvents is mandatory, as well as a clean-up step before quantification of AFM1.

Many biosensors have been shown to have high sensitivity and specificity, enabling rapid detection of a wide range of analytes in complex samples. However, their use for the determination of AFM1 in milk in quality control laboratories and directly on farms or milk collection centers is still limited due to their unreliability. To date, no official method based on biosensors has been recognized and adopted as reference method. However, biosensors have been shown to have potential application for on-site measurements, although at present only one biosensor has been developed, combined with a portable glucose-meter, for the determination of AFM1 in whole milk at levels lower than EU regulatory limits [51]. More efforts should be made to adapt the current developed biosensors to portable devices.

In silico studies on binding mechanisms and the use of artificial intelligence (AI) and machine learning with neural networks could further optimize antibody/aptamer selection and splitting strategies in order to design effective biosensors to be used for the detection of small molecules in complex matrices. The next generation of biosensors based on innovative nanostructures to increase sensitivity and stability could, in the near future, lead to the development of reliable devices able to compete with other currently available analytical methods.

## 5. Patents

A method for detecting mycotoxins in milk, derivates and dairy products. Inventors: Di Giovanni, Stefano; Zambrini, Angelo Vittorio; D’Auria, Sabato. Publication Number WO/2015/063716. Publication Date 7 May 2015.

## Figures and Tables

**Table 3:** Advantages and disadvantages of aptamer- and antibody-based biosensors/assays.

	Antibody-Based Biosensors/Assays	Aptamer-Based Biosensors/Assays
Advantages	Quick and Continuous Measurements High Specificity Rapid Response High/Good Sensitivity Minimal Reagent Usage Cost-Effectiveness Portability and Ease of Use	Low Immunogenicity Low Toxicity Low Production Cost Ease of Production Ease of Modification High Affinity Low/Good Sensitivity * High Chemical and Thermal Stability Good Specificity
Disadvantages	Sensitive to Organic Solvent Sensor Regeneration Problems	Low/Good Sensitivity *

* depending on the target molecule.

## Data Availability

No new data were created or analyzed in this study.
